# P-1211. Early Infections Predict Weight Gain and Obesity in US Children

**DOI:** 10.1093/ofid/ofae631.1393

**Published:** 2025-01-29

**Authors:** Zackary W Taylor, Jane Lin

**Affiliations:** Southern California Permanente Medical Group, Los Angeles, California; Kaiser Permenente - Division of Biostatistics, Pasadena, California

## Abstract

**Background:**

An unexplained link between early viral infection and later weight gain has been observed in the US and internationally. In our population of children at Kaiser Permanente Southern California, we seek to confirm this association and determine the magnitude of the absolute weight increase.

**Figure:**
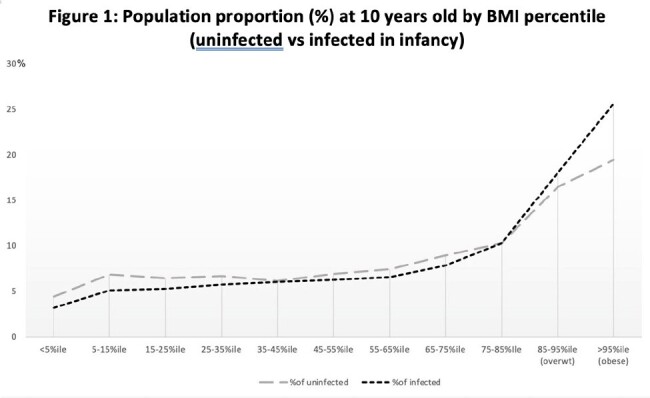

**Methods:**

In a retrospective cohort of children born 2008 – 2012, frequency of infection in infancy (≤12m), timing of first infection (< 6m vs 6-12m), and antibiotic use, class, timing, and frequency were assessed relative to absolute weight and body mass index (BMI) at age 4-6 and 10 years. Patients with low birthweight and prematurity were excluded. Maternal BMI, breastfeeding, birthweight, delivery type, and race/ethnicity were included in multivariable regression; height was also included when absolute weight was the outcome variable. Weight outcomes were compared in uninfected vs infected without antibiotic treatment in infancy (IWOT); and IWOT vs those infected who received antibiotic treatment (IWAT), using logistic regression analysis for obesity outcomes and linear regression for absolute weight.

**Figure:**
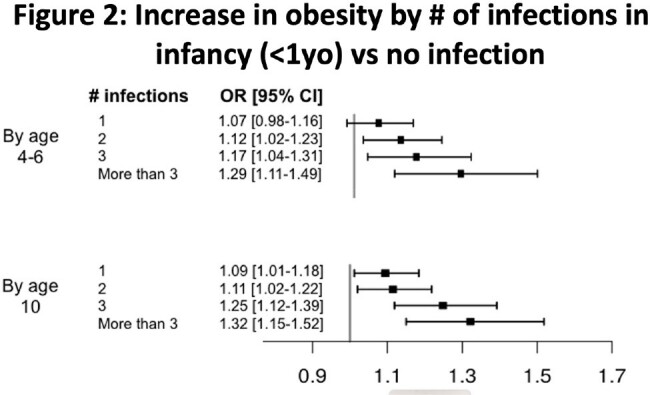

**Results:**

We identified N=66,687 children at 4-6y and N= 46,462 at 10y for analysis. Of the latter, 7,918 had been uninfected, 18,144 had IWOT, and 20,400 had IWAT. The IWOT group had higher rates of obesity vs uninfected (24% vs 19% at 10y; see figure 1), reflected in multivariable analysis at 4-6y (OR 1.11 [1.03-1.2]; p=0.004) and 10y (OR 1.14 [1.06-1.22]; p < 0.001), in a "dose-dependent" manner (Figure 2). The corresponding absolute weight increase (+0.55 kg [+0.3-0.8] at 10y; p< 0.001) was also dose-dependent (Figure 3). Compared to untreated infections, the additional contribution of antibiotic use to future obesity was minimal (OR 1.05 [0.99-1.1]; p=0.09) and not dose-dependent. Of the 10 year olds, most had upper respiratory infections (27,562 patients had at least one URI; 59%), ear infections (16,404 patients; 35%), or infections without a specified site (including viral syndromes; 11,822; 25%) in infancy.

**Figure:**
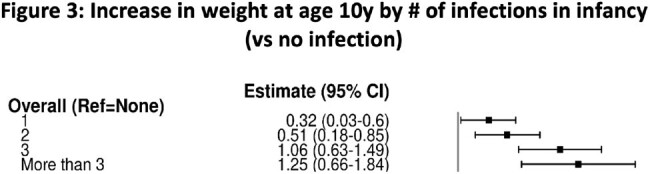

**Conclusion:**

A significant (14% at 10y) increase in obesity after early infection is confirmed, with a small (+0.55kg) corresponding increase in average weight. Prospective study in specific subgroups or with specific viruses of interest would be warranted.

**Disclosures:**

**All Authors**: No reported disclosures

